# Supplementary material for: HOPS: a quantitative score reveals pervasive horizontal pleiotropy in human genetic variation is driven by extreme polygenicity of human traits and diseases
Source: Genome Biol. 2019 Oct 25;20:222. doi: 10.1186/s13059-019-1844-7 (PMC6815001; doi:10.1186/s13059-019-1844-7)
Supplement: Supplementary file 1 — Additional file 1. Supplementary Figures S1-S8, and Tables S1-S4. [file 13059_2019_1844_MOESM1_ESM.docx]

Supplemental Information: Pervasive horizontal pleiotropy in human genetic variation is driven by extreme polygenicity of human traits and diseases

## Power to detect pleiotropy in simulations

**Fig. S1: Effect of LD quantile on two-component pleiotropy score.** Dots show simulated $P_{m}$ (left) and $P_{n}$ (right), both with and without LD-correction, stratified by the simulated level of LD. In both cases, the highest quantile of LD shows substantially inflated pleiotropy score without LD-correction, but applying the LD correction reduces the pleiotropy score to the level seen when there is no LD.

**Fig. S2: Simulation study showing power of two-component pleiotropy score with increased number of traits effected by pleiotropy.**

Black line shows 80% power. Simulations were run for both $P_{m}^{LD}$ (left) and $P_{n}^{LD}$ (right), and both without correction for polygenicity (a,c) and with the correction (b,d), with per-variant heritability ranging from 0.0002 to 0.2, proportion of non-pleiotropic causal loci ranging from 0 to 1%, and proportion of pleiotropic causal loci ranging from 0.1% to 1%. This figure shows a scenario where each pleiotropic variant affects 20 traits, as opposed to the main text figure (**Figure 2**) which shows each pleiotropic variant affecting 10 traits.

**Fig. S3: Simulation study showing power of two-component pleiotropy score with correlation structure.**

Black line shows 80% power. Simulations were run for both $P_{m}^{LD}$ (left) and $P_{m}^{LD}$ (right), and both without correction for polygenicity (a,c) and with the correction (b,d), with per-variant heritability ranging from 0.0002 to 0.2, proportion of non-pleiotropic causal loci ranging from 0 to 1%, and proportion of pleiotropic causal loci ranging from 0.1% to 1%. This figure shows a scenario where traits have correlations between them, as opposed to the main text figure (**Figure 2**) which shows uncorrelated traits.

## Genome-Wide Pleiotropy Study (GWPS) reveals pervasive pleiotropy

Fig. S4: Quantile-quantile plots (Q-Q plot) of the genome-wide pleiotropy study (GWPS) for the 372 medical traits from the UK Biobank.

In each plot the x-axis represent the expected distribution of *P-values*, and the y-axis the observed *P-values* for $P_{m}^{LD}$ (red dots) and $P_{n}^{LD}$ (blue dots). The line shows the observed *P-values* under the null hypothesis. -log_10_ transformed *P-values* are represented.

**Fig. S5: Manhattan plot of the genome-wide pleiotropy study (GWPS) using the two-component pleiotropy score on the 372 heritable medical traits from the UK Biobank.**

The results of the GWPS are provided for the two components of the pleiotropy score, $P_{m}^{LD}$ (top) and $P_{n}^{LD}$ (bottom) on the 372 heritable medical traits from the UK Biobank. Each dot representing a variant is colored by chromosome and ordered by position on the x axis. -log_10_ transformed *P-values* are represented on the y axis. The red line represents the genome-wide significant cut-off of 5 x 10^-8^

**Fig. S6: Scatterplot of the two-component pleiotropy score (left:** $\boldsymbol{P}_{\boldsymbol{m}}^{\boldsymbol{LD}}$**, right:** $\boldsymbol{P}_{\boldsymbol{n}}^{\boldsymbol{LD}}$**) between two decorrelation procedures, using all variants (All) or an independent subset (Pruned) to estimate the phenotype-correlation matrix.**

## Pleiotropy is driven by polygenicity

Fig. S7: Quantile-quantile plots (Q-Q plot) of the genome-wide pleiotropy study (GWPS) for the 372 medical traits from the UK Biobank after applying the correction for polygenicity.

In each plot the x-axis represent the expected distribution of *P-values*, and the y-axis the observed *P-values* for $P_{m}^{P}$ (red dots) and $P_{n}^{P}$ (blue dots). The line shows the observed *P-values* under the null hypothesis. -log_10_ transformed *P-values* are represented.

**Fig. S8: Manhattan plot of the genome-wide pleiotropy study (GWPS) using the two-component polygenicity/LD-corrected pleiotropy score on the 372 heritable medical traits from the UK Biobank after applying the correction for polygenicity.**

The results of the GWPS are provided for the two components of the polygenicity/LD-corrected pleiotropy score, $P_{m}^{P}$ (top) and $P_{n}^{P}$ (bottom) on the 372 heritable medical traits from the UK Biobank. Each dot representing a variant is colored by chromosome and ordered by position on the x axis. -log_10_ transformed *P-values* are represented on the y axis. The red line in panel b represents the genome-wide significant cut-off of 5 x 10^-8^; no variants rise to the level of genome-wide significance in $P_{m}^{P}$ (panel a).

Table S1: Effect of polygenicity on pleiotropy score

|  |  |  |  | **% genome-wide significant variants** | | | **Inflation factor** $\lambda_{GC}$ **of the pleiotropy score** | | | **Mann-Whitney test (greater)** | | | **Mann-Whitney test (less)** | | |
| --- | --- | --- | --- | --- | --- | --- | --- | --- | --- | --- | --- | --- | --- | --- | --- |
| **Range of** $\boldsymbol{h}^{\boldsymbol{2}}$ | **Range of** $\lambda_{GC}^{c}$ | **Average** $\boldsymbol{h}^{\boldsymbol{2}}$ | **# traits** | $\boldsymbol{P}_{\boldsymbol{m}}^{\boldsymbol{LD}}$ | $\boldsymbol{P}_{\boldsymbol{n}}^{\boldsymbol{LD}}$ | $\boldsymbol{P}_{\boldsymbol{m}}^{\boldsymbol{LD}}$ | | $\boldsymbol{P}_{\boldsymbol{n}}^{\boldsymbol{LD}}$ | $\boldsymbol{P}_{\boldsymbol{m}}^{\boldsymbol{LD}}$ | | $\boldsymbol{P}_{\boldsymbol{n}}^{\boldsymbol{LD}}$ | $\boldsymbol{P}_{\boldsymbol{m}}^{\boldsymbol{LD}}$ | | $\boldsymbol{P}_{\boldsymbol{n}}^{\boldsymbol{LD}}$ |  |
| 0.76%-4.3% | 1.029-1.046 | 2.942 | 28 | 0.0010 | 0 | 0.9685 | | 0.6623 | 1 | | 1 | 8.63x10^-19^ | | 0 |  |
| 0.76%-4.3% | 1.046-1.063 | 2.691 | 28 | 0.0012 | 0 | 1.1153 | | 0.6623 | 1.08x10^-291^ | | 1 | 1 | | 0 |  |
| 0.76%-4.3% | 1.063-1.091 | 2.620 | 28 | 0.0116 | 0.0003 | 1.3596 | | 0.6623 | 0 | | 1 | 1 | | 0 |  |
| 0.76%-4.3% | 1.091-1.146 | 2.608 | 28 | 0.0005 | 0 | 1.2836 | | 0.6623 | 0 | | 1 | 1 | | 0 |  |
| 0.76%-4.3% | 1.146-1.251 | 3.548 | 27 | 0.0594 | 0.0005 | 1.6868 | | 0.6623 | 0 | | 1 | 1 | | 3.11x10^-40^ |  |
| 4.3%-7.27% | 1.033-1.053 | 5.364 | 28 | 0.0545 | 0.0001 | 1.2872 | | 0.6623 | 0 | | 1 | 1 | | 0 |  |
| 4.3%-7.27% | 1.053-1.079 | 6.048 | 28 | 0.2564 | 0.0063 | 2.1483 | | 0.6623 | 0 | | 0 | 1 | | 1 |  |
| 4.3%-7.27% | 1.079-1.118 | 5.650 | 28 | 1.1707 | 0.1471 | 5.4103 | | 3.4573 | 0 | | 0 | 1 | | 1 |  |
| 4.3%-7.27% | 1.118-1.273 | 5.505 | 28 | 5.4601 | 0.8052 | 5.9886 | | 3.4573 | 0 | | 0 | 1 | | 1 |  |
| 4.3%-7.27% | 1.273-1.38 | 6.117 | 27 | 5.9312 | 1.2484 | 4.8754 | | 3.4573 | 0 | | 0 | 1 | | 1 |  |
| 7.27%-12.79% | 1.031-1.056 | 9.830 | 28 | 0.0055 | 0 | 1.1411 | | 0.6623 | 0 | | 1 | 1 | | 0 |  |
| 7.27%-12.79% | 1.056-1.093 | 9.448 | 28 | 0.0102 | 0 | 1.2913 | | 0.6623 | 0 | | 1 | 1 | | 0 |  |
| 7.27%-12.79% | 1.093-1.157 | 9.921 | 28 | 0.0065 | 0 | 1.4285 | | 0.6623 | 0 | | 1 | 1 | | 0 |  |
| 7.27%-12.79% | 1.157-1.268 | 9.981 | 28 | 0.0236 | 0.0009 | 1.4573 | | 0.6623 | 0 | | 1 | 1 | | 0 |  |
| 7.27%-12.79% | 1.268-1.587 | 9.824 | 27 | 0.0080 | 0.0008 | 1.6934 | | 0.6623 | 0 | | 0.9996 | 1 | | 0.0004 |  |
| 12.79%-42.19% | 1.028-1.138 | 16.434 | 28 | 0.0231 | 0 | 1.2148 | | 0.6623 | 0 | | 1 | 1 | | 0 |  |
| 12.79%-42.19% | 1.138-1.254 | 22.421 | 28 | 0.0405 | 0.0001 | 1.2222 | | 0.6623 | 0 | | 1 | 1 | | 0 |  |
| 12.79%-42.19% | 1.254-1.415 | 21.446 | 28 | 0.0628 | 0.0020 | 1.4249 | | 0.6623 | 0 | | 1 | 1 | | 0 |  |
| 12.79%-42.19% | 1.415-1.912 | 20.373 | 28 | 0.1056 | 0.0035 | 1.5504 | | 0.6623 | 0 | | 1 | 1 | | 3.51x10^-36^ |  |
| 12.79%-42.19% | 1.912-2.045 | 23.097 | 27 | 0.1506 | 0.0103 | 1.9366 | | 0.6623 | 0 | | 0 | 1 | | 1 |  |

## Genome-wide pleiotropy study identifies novel biological loci

Table S2: Counts of genome-wide significant variants for the pleiotropy score.

|  |  | $P_{n}^{LD}$ | | |
| --- | --- | --- | --- | --- |
|  |  | *P* > 0.05 | 5 x 10^-8^ < *P* < 0.05 | P < 5 x 10^-8^ |
| $P_{m}^{LD}$ | *P* > 0.05 | 272,396 | 33,998 | 0 |
|  | 5 x 10^-8^ < *P* < 0.05 | 113,505 | 265,662 | 354 |
|  | P < 5 x 10^-8^ | 6,529 | 56,027 | 18,587 |

## Pleiotropic loci replicate in independent GWAS datasets

Table S3: Summary of publicly available genome-wide association summary statistic datasets used in the present study.

| Category | Name | Consortium | Link | Reference |
| --- | --- | --- | --- | --- |
| CAD | Total cholesterol | Global Lipids Genetics | [Link](http://csg.sph.umich.edu/abecasis/public/lipids2013/jointGwasMc_TC.txt.gz) | (1) |
| CAD | Low-density lipoprotein cholesterol | Global Lipids Genetics | [Link](http://csg.sph.umich.edu/abecasis/public/lipids2013/jointGwasMc_LDL.txt.gz) | (1) |
| CAD | High-density lipoprotein cholesterol | Global Lipids Genetics | [Link](http://csg.sph.umich.edu/abecasis/public/lipids2013/jointGwasMc_HDL.txt.gz) | (1) |
| CAD | Triglycerides | Global Lipids Genetics | [Link](http://csg.sph.umich.edu/abecasis/public/lipids2013/jointGwasMc_TG.txt.gz) | (1) |
| CAD | Coronary artery disease | CARDIoGRAMplusC4D | [Link](ftp://ftp.sanger.ac.uk/pub/cardiogramplusc4d/2015/cad.additive.Oct2015.pub.zip) | (2) |
| Adiposity | Body mass index | GIANT | [Link](https://www.broadinstitute.org/collaboration/giant/images/1/15/SNP_gwas_mc_merge_nogc.tbl.uniq.gz) | (3) |
| Adiposity | Waist circumference | GIANT | [Link](https://www.broadinstitute.org/collaboration/giant/images/5/57/GIANT_2015_WC_COMBINED_EUR.txt.gz) | (4) |
| Adiposity | Waist circumference (Adj BMI) | GIANT | [Link](https://www.broadinstitute.org/collaboration/giant/images/7/73/GIANT_2015_WCadjBMI_COMBINED_EUR.txt.gz) | (4) |
| Adiposity | Hip circumference | GIANT | [Link](https://www.broadinstitute.org/collaboration/giant/images/e/e4/GIANT_2015_HIP_COMBINED_EUR.txt.gz) | (4) |
| Adiposity | Hip circumference (Adj BMI) | GIANT | [Link](https://www.broadinstitute.org/collaboration/giant/images/5/52/GIANT_2015_HIPadjBMI_COMBINED_EUR.txt.gz) | (4) |
| Adiposity | Waist hip ratio | GIANT | [Link](https://www.broadinstitute.org/collaboration/giant/images/5/54/GIANT_2015_WHR_COMBINED_EUR.txt.gz) | (4) |
| Adiposity | Waist hip ratio (Adj BMI) | GIANT | [Link](https://www.broadinstitute.org/collaboration/giant/images/e/eb/GIANT_2015_WHRadjBMI_COMBINED_EUR.txt.gz) | (4) |
| Adiposity | Height | GIANT | [Link](https://www.broadinstitute.org/collaboration/giant/images/0/01/GIANT_HEIGHT_Wood_et_al_2014_publicrelease_HapMapCeuFreq.txt.gz) | (5) |
| Adiposity | Adiponectin | ADIPOGen | [Link](https://www.mcgill.ca/genepi/files/genepi/adipogen.discovery.eur_.meta_.public.release.part1_.txt) | (6) |
| T2D | Type-2 diabetes | DIAGRAM | [Link](http://diagram-consortium.org/downloads.html) | (7) |
| T2D | Systolic blood pressure | ICBP | Request | (8) |
| T2D | Diastolic blood pressure | ICBP | Request | (8) |
| T2D | Fasting insulin | MAGIC | [Link](ftp://ftp.sanger.ac.uk/pub/magic/MAGIC_ln_FastingInsulin.txt) | (9) |
| T2D | HOMA IR | MAGIC | [Link](ftp://ftp.sanger.ac.uk/pub/magic/MAGIC_ln_HOMA-IR.txt) | (10) |
| T2D | Fasting insulin interaction | MAGIC | [Link](ftp://ftp.sanger.ac.uk/pub/magic/MAGIC_Manning_et_al_lnFastingInsulin_Interaction.txt.gz) | (11) |
| T2D | Fasting proinsulin | MAGIC | [Link](ftp://ftp.sanger.ac.uk/pub/magic/MAGIC_ln_fastingProinsulin.txt.gz) | (9) |
| T2D | Area under the curve - insulin/glucose | MAGIC | [Link](ftp://ftp.sanger.ac.uk/pub/magic/MAGIC_InsulinSecretion_data_release_May2014.zip) | (12) |
| T2D | Area under the curve - insulin | MAGIC | [Link](ftp://ftp.sanger.ac.uk/pub/magic/MAGIC_InsulinSecretion_data_release_May2014.zip) | (12) |
| T2D | Corrected insulin response | MAGIC | [Link](ftp://ftp.sanger.ac.uk/pub/magic/MAGIC_InsulinSecretion_data_release_May2014.zip) | (12) |
| T2D | Corrected insulin response / insulin sensitivity | MAGIC | [Link](ftp://ftp.sanger.ac.uk/pub/magic/MAGIC_InsulinSecretion_data_release_May2014.zip) | (12) |
| T2D | DI | MAGIC | [Link](ftp://ftp.sanger.ac.uk/pub/magic/MAGIC_InsulinSecretion_data_release_May2014.zip) | (12) |
| T2D | Incretin 30 min | MAGIC | [Link](ftp://ftp.sanger.ac.uk/pub/magic/MAGIC_InsulinSecretion_data_release_May2014.zip) | (12) |
| T2D | Insulin 30 min (Adj BMi) | MAGIC | [Link](ftp://ftp.sanger.ac.uk/pub/magic/MAGIC_InsulinSecretion_data_release_May2014.zip) | (12) |
| T2D | Insulin 30 min | MAGIC | [Link](ftp://ftp.sanger.ac.uk/pub/magic/MAGIC_InsulinSecretion_data_release_May2014.zip) | (12) |
| T2D | Insulin sensitivity | MAGIC | [Link](ftp://ftp.sanger.ac.uk/pub/magic/MAGIC_InsulinSecretion_data_release_May2014.zip) | (12) |
| T2D | Fasting glucose | MAGIC | [Link](ftp://ftp.sanger.ac.uk/pub/magic/MAGIC_ln_FastingGlucose.txt) | (9) |
| T2D | HOMA B | MAGIC | [Link](ftp://ftp.sanger.ac.uk/pub/magic/MAGIC_ln_HOMA-B.txt) | (10) |
| T2D | Fasting glucose interaction | MAGIC | [Link](ftp://ftp.sanger.ac.uk/pub/magic/MAGIC_Manning_et_al_FastingGlucose_Interaction.txt.gz) | (11) |
| T2D | Two-hour glucose | MAGIC | [Link](ftp://ftp.sanger.ac.uk/pub/magic/MAGIC_2hrGlucose_AdjustedForBMI.txt) | (9) |
| T2D | Glycated Hemoglobin A1C | MAGIC | [Link](ftp://ftp.sanger.ac.uk/pub/magic/MAGIC_HbA1C.txt.gz) | (9) |
| CKD | Chronic kidney disease | CKDGEN | [Link](http://ckdgen.imbi.uni-freiburg.de/files/Pattaro2016/formatted_round3meta_CKD_overall_IV_2GC_b36_MAFget005_Nget50_20120725_b37.csv.gz) | (13) |
| CKD | Estimated glomerular filtration rate (creatinine) | CKDGEN | [Link](http://ckdgen.imbi.uni-freiburg.de/files/Pattaro2016/formatted_round3meta_eGFRcrea_overall_IV_2GC_b36_MAFget005_Nget50_20120705_b37.csv.gz) | (13) |
| CKD | Estimated glomerular filtration rate (cystatin) | CKDGEN | [Link](http://ckdgen.imbi.uni-freiburg.de/files/Pattaro2016/formatted_round3meta_eGFRcys_overall_IV_2GC_b36_MAFget005_Nget50_20120725_b37.csv.gz) | (13) |
| CKD | Urate | GUGC | Request | (14) |
| Inflammatory Markers | C-reactive protein | CHARGE | Request | (15) |
| Inflammatory Markers | Lipoprotein a | Framingham Heart Study | Request |  |
| Inflammatory Diseases | Rheumatoid arthritis | - | [Link](http://plaza.umin.ac.jp/~yokada/datasource/files/GWASMetaResults/RA_GWASmeta_European_v2.txt.gz) | (16) |
| Inflammatory Diseases | Crohn's disease | IIBDGC | [Link](ftp://ftp.sanger.ac.uk/pub/consortia/ibdgenetics/cd-meta.txt.gz) | (17) |
| Inflammatory Diseases | Inflammatory bowel disease | IIBDGC | [Link](ftp://ftp.sanger.ac.uk/pub/consortia/ibdgenetics/iibdgc-trans-ancestry-summary-stats.tar) | (17) |
| Inflammatory Diseases | Ulcerative colitis | IIBDGC | [Link](ftp://ftp.sanger.ac.uk/pub/consortia/ibdgenetics/ucmeta-sumstats.txt.gz) | (17) |
| Inflammatory Diseases | Gout | GUGC | Request | (14) |
| Inflammatory Diseases | Alzheimer's disease | IGAP | [Link](http://www.pasteur-lille.fr/en/recherche/u744/igap/igap_download.php) | (18) |
| Psychiatric Disorders | Bipolar disorder | PGC | [Link](https://www.med.unc.edu/pgc/results-and-downloads/data-use-agreement-forms/BIP_data_download_agreement) | (19) |
| Psychiatric Disorders | Schizophrenia | PGC | [Link](https://www.med.unc.edu/pgc/results-and-downloads/data-use-agreement-forms/SCZSNP_data_download_agreement) | (20) |
| Psychiatric Disorders | Attention deficit hyperactivity disorder | PGC | [Link](https://www.med.unc.edu/pgc/results-and-downloads/data-use-agreement-forms/CrossADHD_data_download_agreement) | (21) |
| Psychiatric Disorders | Autism spectrum disorder | PGC | [Link](https://www.med.unc.edu/pgc/results-and-downloads/data-use-agreement-forms/CrossASD_data_download_agreement) | (22) |
| Psychiatric Disorders | Eating disorder | PGC | [Link](https://www.med.unc.edu/pgc/results-and-downloads/data-use-agreement-forms/ED_data_download_agreement) | (22) |
| Psychiatric Disorders | Major depressive disorder | PGC | [Link](https://www.med.unc.edu/pgc/results-and-downloads/data-use-agreement-forms/MDD_data_download_agreement) | (23) |
| Brain Volume | Intracranial volume | ENIGMA | [Link](http://enigma.ini.usc.edu/research/download-enigma-gwas-results/data-agreement-for-enigma2-download/) | (24) |
| Brain Volume | Mean accumbens | ENIGMA | [Link](http://enigma.ini.usc.edu/research/download-enigma-gwas-results/data-agreement-for-enigma2-download/) | (24) |
| Brain Volume | Mean amygdala | ENIGMA | [Link](http://enigma.ini.usc.edu/research/download-enigma-gwas-results/data-agreement-for-enigma2-download/) | (24) |
| Brain Volume | Mean caudate | ENIGMA | [Link](http://enigma.ini.usc.edu/research/download-enigma-gwas-results/data-agreement-for-enigma2-download/) | (24) |
| Brain Volume | Mean hippocampus | ENIGMA | [Link](http://enigma.ini.usc.edu/research/download-enigma-gwas-results/data-agreement-for-enigma2-download/) | (24) |
| Brain Volume | Mean pallidum | ENIGMA | [Link](http://enigma.ini.usc.edu/research/download-enigma-gwas-results/data-agreement-for-enigma2-download/) | (24) |
| Brain Volume | Mean putamen | ENIGMA | [Link](http://enigma.ini.usc.edu/research/download-enigma-gwas-results/data-agreement-for-enigma2-download/) | (24) |
| Brain Volume | Mean thalamus | ENIGMA | [Link](http://enigma.ini.usc.edu/research/download-enigma-gwas-results/data-agreement-for-enigma2-download/) | (24) |
| Red blood cell | Hemoglobin | HaemGen | [Link](https://ega-archive.org/studies/EGAS00000000132) | (25) |
| Red blood cell | Mean cell hemoglobin | HaemGen | [Link](https://ega-archive.org/studies/EGAS00000000132) | (25) |
| Red blood cell | Mean corpuscular hemoglobin concentration | HaemGen | [Link](https://ega-archive.org/studies/EGAS00000000132) | (25) |
| Red blood cell | Mean cell volume | HaemGen | [Link](https://ega-archive.org/studies/EGAS00000000132) | (25) |
| Red blood cell | Packed cell volume | HaemGen | [Link](https://ega-archive.org/studies/EGAS00000000132) | (25) |
| Red blood cell | Red blood cell count | HaemGen | [Link](https://ega-archive.org/studies/EGAS00000000132) | (25) |
| Reproductive Genetics | Age Menarche | ReproGen | [Link](http://www.reprogen.org/Menarche_Nature2014_GWASMetaResults_17122014.zip) | (26) |
| Reproductive Genetics | Age Menopause | ReproGen | [Link](http://www.reprogen.org/Menopause_HapMap2_DayNG2015_18112015.txt.gz) | (27) |
| Early Birth | Birth length | EGG | [Link](http://egg-consortium.org/downloads/EGG-GWAS-BL.txt.gz) | (28) |
| Early Birth | Birth weight | EGG | [Link](http://egg-consortium.org/downloads/EGG_BW2_DISCOVERY.txt.gz) | (29) |
| Early Birth | Childhood BMI | EGG | [Link](http://egg-consortium.org/downloads/EGG_BMI_HapMap_DISCOVERY.txt.gz) | (30) |
| Early Birth | Childhood obesity | EGG | [Link](http://egg-consortium.org/downloads/EGG_Obesity_Meta_Analysis_1.txt.gz) | (31) |
| Early Birth | Head circumference | EGG | [Link](http://egg-consortium.org/downloads/EGG_HC_DISCOVERY.txt.gz) | (32) |
| Early Birth | Pubertal growth | EGG | [Link](http://egg-consortium.org/downloads/Pubertal_growth_10F_12M_combined.txt.gz) | (33) |
| Early Birth | Total growth across pubertal growth period | EGG | [Link](http://egg-consortium.org/downloads/Pubertal_growth_PGF_PGM_combined.txt.gz) | (33) |
| Early Birth | Total growth in late adolescent | EGG | [Link](http://egg-consortium.org/downloads/Pubertal_growth_PTF_PTM_combined.txt.gz) | (33) |
| Social Science | Number of years of education | SSGAC | [Link](http://ssgac.org/documents/SSGAC_Rietveld2013.zip) | (34) |
| Smoking History | Number of cigarettes per day | TGC | [Link](https://www.med.unc.edu/pgc/files/resultfiles/tag.cpd.tbl.gz) | (35) |
| Smoking History | Age of onset smoking | TGC | [Link](https://www.med.unc.edu/pgc/files/resultfiles/tag.logonset.tbl.gz) | (35) |
| Smoking History | Ever smoked | TGC | [Link](https://www.med.unc.edu/pgc/files/resultfiles/tag.evrsmk.tbl.gz) | (35) |
| Smoking History | Former smoker | TGC | [Link](https://www.med.unc.edu/pgc/files/resultfiles/tag.former.tbl.gz) | (35) |
| Metabolites | 453 metabolites | Shin et al. | [Link](http://mips.helmholtz-muenchen.de/proj/GWAS/gwas/index.php?task=download) | (36) |
| UK biobank | 2,419 phenotypes | Neale lab | [Link](http://www.nealelab.is/blog/2017/7/19/rapid-gwas-of-thousands-of-phenotypes-for-337000-samples-in-the-uk-biobank) |  |

## Pleiotropy is correlated with specific complex traits and diseases

Table S4: Sensitivity analysis of the pleiotropy score according to the maximum sample size of summary statistics datasets.

|  | **Maximum sample size** | $\lambda_{GC}$ | **Correlation with** $P_{m}^{LD}$ | **Correlation with** $P_{n}^{LD}$ |
| --- | --- | --- | --- | --- |
| BMI (All GIANT) | 339,224 | 1.171 | 0.192 | 0.201 |
| BMI (Men) | 152,893 | 1.072 | 0.138 | 0.141 |
| BMI (Women) | 171,976 | 1.073 | 0.147 | 0.157 |
| CAD (CARDIoGRAM) | 84,264 | 1.105 | 0.071 | 0.070 |
| CAD (CARDIoGRAM+C4D) | 184,305 | 1.062 | 0.124 | 0.119 |

BMI: Body mass index; CAD: Coronary artery disease.

# References

1.	Global Lipids Genetics Consortium, Willer CJ, Schmidt EM, Sengupta S, Peloso GM, Gustafsson S, et al. Discovery and refinement of loci associated with lipid levels. Nat Genet. 2013 Nov;45(11):1274–83.

2.	the CARDIoGRAMplusC4D Consortium. A comprehensive 1000 Genomes-based genome-wide association meta-analysis of coronary artery disease. Nat Genet. 2015 Oct;47(10):1121–30.

3.	Locke AE, Kahali B, Berndt SI, Justice AE, Pers TH, Day FR, et al. Genetic studies of body mass index yield new insights for obesity biology. Nature. 2015 Feb 12;518(7538):197–206.

4.	Shungin D, Winkler TW, Croteau-Chonka DC, Ferreira T, Locke AE, Mägi R, et al. New genetic loci link adipose and insulin biology to body fat distribution. Nature. 2015 Feb 12;518(7538):187–96.

5.	Wood AR, Esko T, Yang J, Vedantam S, Pers TH, Gustafsson S, et al. Defining the role of common variation in the genomic and biological architecture of adult human height. Nat Genet. 2014 Nov;46(11):1173–86.

6.	Dastani Z, Hivert M-F, Timpson N, Perry JRB, Yuan X, Scott RA, et al. Novel loci for adiponectin levels and their influence on type 2 diabetes and metabolic traits: a multi-ethnic meta-analysis of 45,891 individuals. PLoS Genet. 2012;8(3):e1002607.

7.	Morris AP, Voight BF, Teslovich TM, Ferreira T, Segrè AV, Steinthorsdottir V, et al. Large-scale association analysis provides insights into the genetic architecture and pathophysiology of type 2 diabetes. Nat Genet. 2012 Sep;44(9):981–90.

8.	International Consortium for Blood Pressure Genome-Wide Association Studies, Ehret GB, Munroe PB, Rice KM, Bochud M, Johnson AD, et al. Genetic variants in novel pathways influence blood pressure and cardiovascular disease risk. Nature. 2011 Sep 11;478(7367):103–9.

9.	Scott RA, Lagou V, Welch RP, Wheeler E, Montasser ME, Luan J, et al. Large-scale association analyses identify new loci influencing glycemic traits and provide insight into the underlying biological pathways. Nat Genet. 2012 Sep;44(9):991–1005.

10.	Dupuis J, Langenberg C, Prokopenko I, Saxena R, Soranzo N, Jackson AU, et al. New genetic loci implicated in fasting glucose homeostasis and their impact on type 2 diabetes risk. Nat Genet. 2010 Feb;42(2):105–16.

11.	Manning AK, LaValley M, Liu C-T, Rice K, An P, Liu Y, et al. Meta-analysis of Gene-Environment interaction: joint estimation of SNP and SNP×Environment regression coefficients. Genet Epidemiol. 2011 Jan;35(1):11–8.

12.	Prokopenko I, Poon W, Mägi R, Prasad B R, Salehi SA, Almgren P, et al. A central role for GRB10 in regulation of islet function in man. PLoS Genet. 2014 Apr;10(4):e1004235.

13.	Pattaro C, Teumer A, Gorski M, Chu AY, Li M, Mijatovic V, et al. Genetic associations at 53 loci highlight cell types and biological pathways relevant for kidney function. Nat Commun. 2016 Jan 21;7:10023.

14.	Köttgen A, Albrecht E, Teumer A, Vitart V, Krumsiek J, Hundertmark C, et al. Genome-wide association analyses identify 18 new loci associated with serum urate concentrations. Nat Genet. 2013 Feb;45(2):145–54.

15.	Dehghan A, Dupuis J, Barbalic M, Bis JC, Eiriksdottir G, Lu C, et al. Meta-analysis of genome-wide association studies in >80 000 subjects identifies multiple loci for C-reactive protein levels. Circulation. 2011 Feb 22;123(7):731–8.

16.	Okada Y, Wu D, Trynka G, Raj T, Terao C, Ikari K, et al. Genetics of rheumatoid arthritis contributes to biology and drug discovery. Nature. 2014 Feb 20;506(7488):376–81.

17.	Liu JZ, van Sommeren S, Huang H, Ng SC, Alberts R, Takahashi A, et al. Association analyses identify 38 susceptibility loci for inflammatory bowel disease and highlight shared genetic risk across populations. Nat Genet. 2015 Sep;47(9):979–86.

18.	Lambert JC, Ibrahim-Verbaas CA, Harold D, Naj AC, Sims R, Bellenguez C, et al. Meta-analysis of 74,046 individuals identifies 11 new susceptibility loci for Alzheimer’s disease. Nat Genet. 2013 Dec;45(12):1452–8.

19.	Psychiatric GWAS Consortium Bipolar Disorder Working Group. Large-scale genome-wide association analysis of bipolar disorder identifies a new susceptibility locus near ODZ4. Nat Genet. 2011 Sep 18;43(10):977–83.

20.	Schizophrenia Working Group of the Psychiatric Genomics Consortium. Biological insights from 108 schizophrenia-associated genetic loci. Nature. 2014 Jul 24;511(7510):421–7.

21.	Neale BM, Medland SE, Ripke S, Asherson P, Franke B, Lesch K-P, et al. Meta-analysis of genome-wide association studies of attention-deficit/hyperactivity disorder. J Am Acad Child Adolesc Psychiatry. 2010 Sep;49(9):884–97.

22.	Cross-Disorder Group of the Psychiatric Genomics Consortium. Identification of risk loci with shared effects on five major psychiatric disorders: a genome-wide analysis. Lancet. 2013 Apr 20;381(9875):1371–9.

23.	Major Depressive Disorder Working Group of the Psychiatric GWAS Consortium, Ripke S, Wray NR, Lewis CM, Hamilton SP, Weissman MM, et al. A mega-analysis of genome-wide association studies for major depressive disorder. Mol Psychiatry. 2013 Apr;18(4):497–511.

24.	Hibar DP, Stein JL, Renteria ME, Arias-Vasquez A, Desrivières S, Jahanshad N, et al. Common genetic variants influence human subcortical brain structures. Nature. 2015 Apr 9;520(7546):224–9.

25.	van der Harst P, Zhang W, Mateo Leach I, Rendon A, Verweij N, Sehmi J, et al. Seventy-five genetic loci influencing the human red blood cell. Nature. 2012 Dec 20;492(7429):369–75.

26.	Perry JRB, Day F, Elks CE, Sulem P, Thompson DJ, Ferreira T, et al. Parent-of-origin-specific allelic associations among 106 genomic loci for age at menarche. Nature. 2014 Oct 2;514(7520):92–7.

27.	Stolk L, Perry JRB, Chasman DI, He C, Mangino M, Sulem P, et al. Meta-analyses identify 13 loci associated with age at menopause and highlight DNA repair and immune pathways. Nat Genet. 2012 Jan 22;44(3):260–8.

28.	van der Valk RJP, Kreiner-Møller E, Kooijman MN, Guxens M, Stergiakouli E, Sääf A, et al. A novel common variant in DCST2 is associated with length in early life and height in adulthood. Hum Mol Genet. 2015 Feb 15;24(4):1155–68.

29.	Horikoshi M, Beaumont RN, Day FR, Warrington NM, Kooijman MN, Fernandez-Tajes J, et al. Genome-wide associations for birth weight and correlations with adult disease. Nature. 2016 Oct 13;538(7624):248–52.

30.	Felix JF, Bradfield JP, Monnereau C, van der Valk RJP, Stergiakouli E, Chesi A, et al. Genome-wide association analysis identifies three new susceptibility loci for childhood body mass index. Hum Mol Genet. 2016 Jan 15;25(2):389–403.

31.	Bradfield JP, Taal HR, Timpson NJ, Scherag A, Lecoeur C, Warrington NM, et al. A genome-wide association meta-analysis identifies new childhood obesity loci. Nat Genet. 2012 May;44(5):526–31.

32.	Taal HR, St Pourcain B, Thiering E, Das S, Mook-Kanamori DO, Warrington NM, et al. Common variants at 12q15 and 12q24 are associated with infant head circumference. Nat Genet. 2012 Apr 15;44(5):532–8.

33.	Cousminer DL, Berry DJ, Timpson NJ, Ang W, Thiering E, Byrne EM, et al. Genome-wide association and longitudinal analyses reveal genetic loci linking pubertal height growth, pubertal timing and childhood adiposity. Hum Mol Genet. 2013 Jul 1;22(13):2735–47.

34.	Okbay A, Beauchamp JP, Fontana MA, Lee JJ, Pers TH, Rietveld CA, et al. Genome-wide association study identifies 74 loci associated with educational attainment. Nature. 2016 26;533(7604):539–42.

35.	Tobacco and Genetics Consortium. Genome-wide meta-analyses identify multiple loci associated with smoking behavior. Nat Genet. 2010 May;42(5):441–7.

36.	Shin S-Y, Fauman EB, Petersen A-K, Krumsiek J, Santos R, Huang J, et al. An atlas of genetic influences on human blood metabolites. Nat Genet. 2014 Jun;46(6):543–50.
