# Supplementary material for: HOPS: a quantitative score reveals pervasive horizontal pleiotropy in human genetic variation is driven by extreme polygenicity of human traits and diseases
Source: Genome Biol. 2019 Oct 25;20:222. doi: 10.1186/s13059-019-1844-7 (PMC6815001; doi:10.1186/s13059-019-1844-7)
Supplement: Supplementary file 4 — Additional file 4. Review history. [file 13059_2019_1844_MOESM4_ESM.docx]

Review History

**First round of review**

**Reviewer 1**

**Are you able to assess all statistics in the manuscript, including the appropriateness of statistical tests used?**

No, I do not feel adequately qualified to assess the statistics.

**Comments to author:**

The paper by Jordan et al deals with an important question regarding the genetic bases of most human traits. That is, to what extent are the underlying genetic models motivated by the same genetic loci even in the absence of clear vertical biological relationships. In the current world of Mendelian Randomization this is important. As noted by the authors it is also important in regard to our understanding of how poly- or omnigenic many traits are. The results of this paper - perhaps not surprisingly - indicate that the genome is highly pleiotropic and by this I mean horizontal pleiotropy.

This is a very complex paper that I think will require careful explanation of the methods underlying theory in a supplement as well as a broader discussion of the implications of the results.

Although the topic is important and timely there are several things that are not completely clear to me.

1) To what extent does the whitening actually remove only vertically correlated traits. For example, if there is true horizontal pleiotropy it is possible if effect sizes are the same and relatively strong both traits would whitening remove the them and therefore underestimate the extent of pleiotropy.

2) Two measures of pleiotropy are measured number and effect - the results are not identical. It is not clear what the implications of these differences are. I would like to see the authors discuss the two measures in terms of what they are actually illustrating.

3) In the discussion there is a statement about the traits that appear to be highly pleiotropic - e.g., BMI, height, schizophrenia. Glucose metabolism does not fall into this category and I find that surprising as diabetes (presumably glucose metabolism) is an extremely complex trait that needs to be robust. It is argued this is due to smaller sample size of datasets but I am not convinced by this argument.

4) In discussing genetic architecture of traits, it appears the authors definition only revolves around number of traits and genes. This is not the complete definition of architecture that can involve the relationship of effects across loci (epstasis). Does the current work have any relevance to this?

Minor Figure S7 has no redline as described in the legend.

**Reviewer 2**

**Are you able to assess all statistics in the manuscript, including the appropriateness of statistical tests used?**

There are no statistics in the manuscript.

**Comments to author:**

Modeling the genetic architecture of complex phenotypes is of fundamental importance to many fields of biology and biomedicine. Pleiotropy is recognized as one of the important components that constitute trait genetic architecture. In this manuscript, the authors proposed a framework for dissecting one of pleiotropy types, horizontal pleiotropy. The manuscript was clearly written, and the authors presented a significant advance in quantitative genetics. I recommend publication after minor revisions as follows:

(1)	The authors define the horizontal pleiotropy to mean that a genetic cause influences multiple traits, without one trait being mediated by another (page 3, line 49). I think this is imprecise. Since different traits are controlled by the same variants, they may be correlated. In reality, no trait is in isolation, rather it mediates or is mediated by others. Or, does the author mean that traits are not correlated before the same variant is at play? It is hard to justify.

(2)	The authors mentioned "independent traits" in many places. As stated above, independent traits may not have the common genetic basis.

(3)	In the simulation studies, I want to see how the extent of LD affects our assessment precision on the pervasion of pleiotropy. You may need to simulate more scenarios by allowing LD to vary in a wide range.

(4)	In addition to LD, the linkage between different loci may also contribute to pleiotropy. How can you study the contribution of the linkage?

(5)	The authors studied 372 phenotypes to test their framework. I am interested to see what these phenotypes are? Are they correlated? It makes more sense to give the correlation table of these phenotypes.

(6)	According to the definition of horizontal pleiotropy, BMI and body height should not be explained to be under the control of horizontal pleiotropy. BMI is a composite trait of height and body mass. The relationship between BMI and height should be explained as something else. Among these 372 traits, how many have relationships like BMI and height?

In summary, this is a nice work and should be published after addressing the above comments.

**Authors Response**

**Point-by-point responses to the reviewers’ comments:**

Response to Reviewer 1’s comments

Reviewer #1: The paper by Jordan et al deals with an important question regarding the genetic bases of most human traits. That is, to what extent are the underlying genetic models motivated by the same genetic loci even in the absence of clear vertical biological relationships. In the current world of Mendelian Randomization this is important. As noted by the authors it is also important in regard to our understanding of how poly- or omnigenic many traits are. The results of this paper - perhaps not surprisingly - indicate that the genome is highly pleiotropic and by this I mean horizontal pleiotropy.

*Authors reply: We thank the Reviewer for their helpful comments.*

This is a very complex paper that I think will require careful explanation of the methods underlying theory in a supplement as well as a broader discussion of the implications of the results.

Although the topic is important and timely there are several things that are not completely clear to me.

1) To what extent does the whitening actually remove only vertically correlated traits. For example, if there is true horizontal pleiotropy it is possible if effect sizes are the same and relatively strong both traits would whitening remove the them and therefore underestimate the extent of pleiotropy.

*Authors reply: We thank the reviewers for pointing out this unclarity in our discussion. This is a central point, and it appeared to be confusing to both reviewers (see Reviewer 2 comment 1 below), so we have added text clarifying it. We have made more explicit the definitions of vertical and horizontal pleiotropy. Vertical pleiotropy is defined by a variant affecting one trait, which then mediates another trait and hence is defined by a relationship between traits. On the other hand, horizontal pleiotropy can exist only on the level of an individual variant or locus. Therefore, any correlation of effect sizes between traits will by definition involve vertical pleiotropy since it’s improbable that multiple genetic variants will have a proportional quantitative effect of trait A and trait B, without one mediating the other. Correlation of effect sizes between traits does not rule out horizontal pleiotropy however. Our whitening approach is based on the idea that any variant-level pleiotropic effects that are still present after removing trait-level correlation are by definition horizontal.*

*In the specific case raised by this reviewer, where a variant has similarly sized strong effects on two traits, the result would depend on the genome-wide correlation between the effect sizes for these two traits. If the effect sizes for the two traits are similar or proportional across all variants in the genome, then this would be considered evidence that the traits are biologically related, and the variant would be scored as having only vertical pleiotropy. If the effect sizes for the two traits are generally unrelated across the entire genome, or if the relationship shown by the specific variant is a significant deviation from the whole-genome (e.g. all variants genome-wide) relationship, the variant would be scored as having horizontal pleiotropy.*

*As part of adding this dedicated discussion of the difference between horizontal and vertical pleiotropy, we have added additional text to clarify this difference, and divided Figure 1 into three figures. Figure 1 contains the former Figures 1a-b, showing the definitions of horizontal and vertical pleiotropy; Figure 2 contains the former Figures 1c-d, showing the contributions of LD and polygenicity to pleiotropy; and Figure 3 is the former Figure 1e, showing the schematic of the method. All other figures have been renumbered accordingly.*

*Manuscript changes: We have added a “Defining pleiotropy” subheading in the Results section, with the following text:*

*We narrowly define the scope of pleiotropy as applying only to genetic variants, and particularly variants investigated as part of GWASs. As effects, we are considering phenotypic outcomes measured by GWASs. By our definition, then, pleiotropy means that one variant shows significant associations across GWASs of multiple traits. We additionally restrict the scope of pleiotropy we are considering to include only horizontal (variant-level) pleiotropy, and to exclude vertical (trait-level) pleiotropy (Figure 1). To elaborate on this distinction, suppose we have identified a variant that influences two different traits, trait A and trait B. In vertical or trait-level pleiotropy, the traits themselves are biologically related, so that the variant’s effect on trait A actually causes the effect on trait B. A key feature of vertical pleiotropy is that two traits that are biologically related should be related regardless of which specific gene or variant is causing the effect. This induces correlation between GWAS effect sizes on the two traits across an entire set of variants. For example, we expect that any variant that increases LDL cholesterol also increases risk of coronary artery disease, because we suspect that it is the increase in LDL cholesterol itself that causes increased disease risk. This results in a correlation between variant effect sizes for LDL cholesterol and coronary artery disease, which has been detected in multiple studies. The methodology of Mendelian Randomization uses this predicted correlation within a given set of variants to formulate a statistical test for causal relationships among traits, which is now widely used for biological discovery. We extend this methodology to use the entire set of SNVs evaluated by GWAS, treating a GWAS-wide correlation between two traits as evidence of a trait-level pleiotropic relationship between these traits.*

*In the case of horizontal or variant-level pleiotropy, an individual variant acts on traits A and B without mirroring any trait-level relationship between them. Unlike vertical pleiotropy, since we are not considering the variant-level effect as evidence of a relationship between the two traits, we cannot detect horizontal pleiotropy by detecting correlations between traits. Instead, each horizontally pleiotropic variant acts by its own unique mechanism. These particular pleiotropic variants, therefore, should show a relationship between the two traits that deviates from the relationship we would infer from the genome-wide correlation of effect sizes between them. This deviation from the correlation between traits is not a prediction of any kind of model of pleiotropy, but simply follows from our definition of the term “horizontal pleiotropy:” any pair of traits whose effect sizes are correlated across all variants is by definition related by vertical pleiotropy, while any variant whose effects on two traits substantially deviate from the trait-level relationship between those traits is by definition exhibiting horizontal pleiotropy.*

2) Two measures of pleiotropy are measured number and effect - the results are not identical. It is not clear what the implications of these differences are. I would like to see the authors discuss the two measures in terms of what they are actually illustrating.

*Authors reply: This is an important omission from the manuscript, and we thank the reviewer for pointing it out. As the Reviewer noted, in principle, the magnitude of a pleiotropic effect and the number of traits affected are conceptually different features of pleiotropy. The fact that they do not meaningfully deviate from each other means that we do not detect either large-effect variants that influence only a small number of traits or small-effect variants that have a tiny effect on a large number of traits. The places where they do differ – most notably, that there are many more significant loci for P_m^LD than P_n^LD, and that this relationship is reversed after applying the polygenicity correction – is informative about the architecture of pleiotropy, and the relationship between the number of traits a variant effects (P_n) and the combined effect size across those traits (P_m). We have edited the manuscript to include these insights.*

*Manuscript changes: We have added the following text to the Results section, under the subheading “A quantitative score for pleiotropy:”*

*In principle these are distinct quantities: the magnitude score P_m measures the total pleiotropic effect size of a variant across all traits, while the number of traits score P_n measures the number of distinct pleiotropic effects a variant has. A variant with a high P_m score and a low P_n score has a large effect spread over a small number of traits; a variant with a low P_m score and a high P_n score has only a minor effect overall, but that effect is spread out across a large number of traits; and a variant with high scores on both components has a large effect that is spread across a large number of traits.*

*We have edited the paragraph in the Results section under the subheading “Genome-wide pleiotropy study identifies novel biological loci.” to include both components of the pleiotropy score instead of just P_n. It now reads, in full:*

*By analogy to standard GWAS, our GWPS methodology can identify individual variants that have a genome-wide significant level of horizontal pleiotropy. Using the LD-corrected magnitude score P_m^LD, we identified 74,335 variants in 8,093 independent loci with a genome-wide significant level of horizontal pleiotropy, while using the LD-corrected number of traits score P_n^LD identified 18,393 variants in 2,859 independent loci with a genome-wide significant level of horizontal pleiotropy, all of which are also identified by the LD-corrected magnitude score P_m^LD (Methods, Supplementary Table 2). Applying the same analysis to the Polygenicity/LD-corrected pleiotropy score, using the Polygenicity/LD-corrected magnitude score P_m^P identified no genome-wide significant loci, but using the Polygenicity/LD-corected number of traits score P_n^P identified 2,674 variants in 432 loci. Strikingly, a majority of loci significant in P_n^LD (1,519 of 2,859) or P_n^P (294 of 432), along with a sizeable minority of loci significant in P_m^LD (2,934 of 8,093), have no entry in the NHGRI-EBI GWAS catalog, meaning that they have never been reported as an associated locus in any published GWAS. These loci represent an under-recognized class of genetic variation that has multiple weak to intermediate effects that are collectively significant, but no specific strong effect on any one particular trait. Functional enrichment analysis on genes near these genome-wide significant loci implicates a wide range of biological functions, including cell adhesion, post-translational modification of proteins, cytoskeleton, transcription factors, and intracellular signaling cascades (Additional File 2). Loci significant in P_n^P show a more focused subset of functions, with a greater role for nuclear proteins regulating transcription and chromatin state, suggesting that these are the functions that exhibit pleiotropy beyond the baseline level induced by polygenicity. The role of these novel loci and these biological processes in human genetics and biology may be a fruitful area for future study, with the potential for biological discovery.*

*We have added the following text to the Discussion section, in the initial listing of our findings:*

*These findings are largely consistent between the magnitude of pleiotropy score P_m and the number of traits score P_n, although we note some differences where some variants are primarily associated with P_m but not P_n. This indicates that these signals are driven by loci that both influence a large number of traits and have relatively large combined effects, and secondarily by loci that have large pleiotropic effects but only influence a handful of traits each, with minimal contribution from loci that influence a large number of traits but have small overall effects.*

*We have added the following text to the Discussion section:*

*These findings are largely consistent between the magnitude of pleiotropy score P_m and the number of traits score P_n, although we note some differences where some variants are primarily associated with P_m^LD but not P_n^LD. This indicates that these signals are driven by loci that both influence a large number of traits and have relatively large combined effects, and secondarily by loci that have large combined effects but only influence a handful of traits each, with minimal contribution from loci that influence a large number of traits but have small combined effects. Conversely, after applying the correction for polygenicity, we only observe variants that are significant for P_n^P, but not for P_m^P. This indicates that, while there do exist pleiotropic master control loci that affect more traits than we would expect from the random overlap of multiple highly polygenic traits, the overall effect of these loci is not noticeably larger than we would expect.*

*…*

*The fact that the magnitude of pleiotropy score P_m and the number of traits score P_n give largely consistent results also supports this model, where a larger biological effect in a given tissue will perturb a greater number of phenotypes relevant to that tissue, although we note that some variants have high magnitude of pleiotropy score P_m and low number of traits score P_n, which may represent a small class of variants that has large biological effects without perturbing a large number of phenotypes.*

3) In the discussion there is a statement about the traits that appear to be highly pleiotropic - e.g., BMI, height, schizophrenia. Glucose metabolism does not fall into this category and I find that surprising as diabetes (presumably glucose metabolism) is an extremely complex trait that needs to be robust. It is argued this is due to smaller sample size of datasets but I am not convinced by this argument.

*Authors reply: The reviewer raises an interesting point about type 2 diabetes and glucose metabolism traits not being in the list of pleiotropy-associated traits, considering that one of our primary conclusions is that pleiotropy is largely driven by complex polygenic traits, and type 2 diabetes is a classic example of such a trait. Study sample size is undoubtedly a factor, as all the most pleiotropy-associated traits are those with the largest study sample size. This would be expected if pleiotropy is mostly driven by variants with small effects on any single trait, which would be easier to detect in a larger sample. Another issue may be that these traits are simply not under tight genetic control. Many of the traits we note as being the least associated with pleiotropic variants are among the least heritable traits we considered: Insulin Sensitivity Index (ISI), area under insulin response curve, and 30-minute insulin level all have estimated h^2<0.05. This, of course, may also be related to sample size. We have added text discussing this.*

*Manuscript changes: We have added text to the Results section, under the subheading “A quantitative score for pleiotropy.” It now reads in total as follows:*

*To characterize the phenotypic associations of these loci, we used our replication dataset of published GWAS summary statistics for 73 human quantitative traits and diseases, plus nine additional traits we excluded from our replication dataset for a total of 82 (Methods). We are not able to compute directly the degree of pleiotropy exhibited by these traits, since our definition of horizontal pleiotropy applies only to individual variants and does not apply to traits. However, we can identify traits whose GWAS variant associations are correlated to our pleiotropy score, which in some sense represents the traits that contribute most to our signal of pervasive horizontal pleiotropy. Figure 6c shows the correlations between our LD-corrected pleiotropy score (P_m^LD and P_n^LD) and the association statistics for these 82 traits and diseases. The most strongly correlated traits were anthropometric traits like body mass index, waist and hip circumference, and height; certain blood lipid levels, including total cholesterol and triglycerides; and schizophrenia. These are all known to be highly polygenic and heterogeneous traits. The least correlated traits include several measurements of insulin sensitivity and glucose response, such as the insulin sensitivity index (ISI), certain features of brain morphology, and the inflammatory biomarker lipoprotein(a). This may be partly due to low sample size of the corresponding GWASs. However, these correlations do not appear to be driven exclusively by sample size: in cases where multiple GWASs for the same trait have been performed on subsamples of the population (for example, males only, female only, and combined), the sample size only marginally affects the correlation (Supplementary Table 4). Another contributing factor may be heritability: height, in particular, is among the most heritable traits we examined, while ISI and the brain morphology features are among the least.*

*We have edited the legend for Figure 6c (formerly Figure 4c) to read as follows:*

*Panel c shows the contribution of pleiotropic variants to 82 complex traits and diseases. Contribution of pleiotropic variants is calculated as the correlation coefficient between the absolute value of Z-scores and the pleiotropy score among variants that are genome-wide significant for the pleiotropy score (P < 5 × 10-8 for P_m^LD and P_n^LD respectively).*

4) In discussing genetic architecture of traits, it appears the authors definition only revolves around number of traits and genes. This is not the complete definition of architecture that can involve the relationship of effects across loci (epstasis). Does the current work have any relevance to this?

*Authors reply: We agree that epistasis can potentially play a role in the genetic architecture of complex traits. In principle, given GWAS summary statistics that included interaction terms between variants, it would be fairly straightforward (though possibly computationally expensive) to incorporate them into our method and produce pleiotropy interaction terms. However, these interaction terms currently are not routinely estimated in GWASs, and can’t be derived from published summary statistics. If there is a case where the effect of a variant depends strongly on its genomic context, we would expect GWAS summary statistics to represent the average effect across all contexts found in the population, and our method would propagate that averaging. This is usually not considered to be a major consideration in GWAS, as interactions between the generally weak-effect common variants assessed by GWASs are assumed to be very weak. We have added a sentence in the manuscript discussing this limitation.*

*Manuscript changes: We have the following sentence to our discussion of the limitations of the method:*

*Third, we assume all genetic effects are additive and independent, and we do not model epistasis or other more complex genetic architectures*.

Minor Figure S7 has no redline as described in the legend.

*Authors reply: The red line is not shown because it was off the scale of the plot, and no loci come close to the level of genome-wide significance. We have rerun the analysis and now find some genome-wide significant loci for P_n^P; we have edited this figure and its legend accordingly.*

*Manuscript changes: We have edited the legend for Figure S7. It now reads:*

*The results of the GWPS are provided for the two components of the polygenicity/LD-corrected pleiotropy score, P_m^P (top) and P_n^P (bottom) on the 372 heritable medical traits from the UK Biobank. Each dot representing a variant is colored by chromosome and ordered by position on the x axis. -log10 transformed P-values are represented on the y axis. The red line in panel b represents the genome-wide significant cut-off of 5 x 10-8; no variants rise to the level of genome-wide significance in P_m^P (panel a).*

Response to Reviewer 2’s comments

Modeling the genetic architecture of complex phenotypes is of fundamental importance to many fields of biology and biomedicine. Pleiotropy is recognized as one of the important components that constitute trait genetic architecture. In this manuscript, the authors proposed a framework for dissecting one of pleiotropy types, horizontal pleiotropy. The manuscript was clearly written, and the authors presented a significant advance in quantitative genetics.

*Authors reply: We thank the Reviewer for their positive comments.*

I recommend publication after minor revisions as follows:

(1) The authors define the horizontal pleiotropy to mean that a genetic cause influences multiple traits, without one trait being mediated by another (page 3, line 49). I think this is imprecise. Since different traits are controlled by the same variants, they may be correlated. In reality, no trait is in isolation, rather it mediates or is mediated by others. Or, does the author mean that traits are not correlated before the same variant is at play? It is hard to justify.

*Authors reply: This is an important question of definitions that warrants clarification. As mentioned above, this point was confusing to both reviewers (see reviewer 1 comment 1 above), so we have added a substantial amount of text attempting to clarify. We thank the reviewers for pointing out this weakness in our discussion. The distinction we want to make between horizontal and vertical pleiotropy is that vertical pleiotropy refers to a relationship between traits due to multiple variants having proportional effects between two traits, while horizontal pleiotropy is a property of a single variant, where a variant influences multiple traits in a way that is not reflected in an overall biological relationship between these traits. There is established theory that treats correlation of effect sizes of variants between traits as evidence of a biological relationship between traits, primarily in the context of Mendelian Randomization. While it is true that any variant that influences multiple traits simultaneously will contribute to a correlation between them, we assume there is a meaningful distinction between a set of traits that is related in a consistent way across all variants and a set of traits whose relationship varies depending on which variants we examine. In the first case, the traits will show a strong correlation overall, and few variants will deviate significantly from that overall correlation; in the second, the traits may show only weak correlation overall, even if there are individual variants that strongly influence both traits.*

*As part of adding this dedicated discussion of the difference between horizontal and vertical pleiotropy, we have added additional text to clarify this difference, and divided Figure 1 into three figures. Figure 1 contains the former Figures 1a-b, showing the definitions of horizontal and vertical pleiotropy; Figure 2 contains the former Figures 1c-d, showing the contributions of LD and polygenicity to pleiotropy; and Figure 3 is the former Figure 1e, showing the schematic of the method. All other figures have been renumbered accordingly.*

*Manuscript changes: We have added a “Defining pleiotropy” subheading in the Results section, with the following text:*

*We narrowly define the scope of pleiotropy as applying only to genetic variants, and particularly variants investigated as part of GWASs. As effects, we are considering phenotypic outcomes measured by GWASs. By our definition, then, pleiotropy means that one variant shows significant associations across GWASs of multiple traits. We additionally restrict the scope of pleiotropy we are considering to include only horizontal (variant-level) pleiotropy, and to exclude vertical (trait-level) pleiotropy (Figure 1). To elaborate on this distinction, suppose we have identified a variant that influences two different traits, trait A and trait B. In vertical or trait-level pleiotropy, the traits themselves are biologically related, so that the variant’s effect on trait A actually causes the effect on trait B. A key feature of vertical pleiotropy is that two traits that are biologically related should be related regardless of which specific gene or variant is causing the effect. This induces correlation between GWAS effect sizes on the two traits across an entire set of variants. For example, we expect that any variant that increases LDL cholesterol also increases risk of coronary artery disease, because we suspect that it is the increase in LDL cholesterol itself that causes increased disease risk. This results in a correlation between variant effect sizes for LDL cholesterol and coronary artery disease, which has been detected in multiple studies. The methodology of Mendelian Randomization uses this predicted correlation within a given set of variants to formulate a statistical test for causal relationships among traits, which is now widely used for biological discovery. We extend this methodology to use the entire set of SNVs evaluated by GWAS, treating a GWAS-wide correlation between two traits as evidence of a trait-level pleiotropic relationship between these traits.*

*In the case of horizontal or variant-level pleiotropy, an individual variant acts on traits A and B without mirroring any trait-level relationship between them. Unlike vertical pleiotropy, since we are not considering the variant-level effect as evidence of a relationship between the two traits, we cannot detect horizontal pleiotropy by detecting correlations between traits. Instead, each horizontally pleiotropic variant acts by its own unique mechanism. These particular pleiotropic variants, therefore, should show a relationship between the two traits that deviates from the relationship we would infer from the genome-wide correlation of effect sizes between them. This deviation from the correlation between traits is not a prediction of any kind of model of pleiotropy, but simply follows from our definition of the term “horizontal pleiotropy:” any pair of traits whose effect sizes are correlated across all variants is by definition related by vertical pleiotropy, while any variant whose effects on two traits substantially deviate from the trait-level relationship between those traits is by definition exhibiting horizontal pleiotropy.*

(2) The authors mentioned "independent traits" in many places. As stated above, independent traits may not have the common genetic basis.

*Authors reply: The reviewer is correct that this is confusing and imprecise terminology, as the “independent traits” we are referring to are independent only in a mathematical sense, and not in the biological sense of having no overlap or interaction. We have changed all uses of this phrase to “decorrelated traits” or “whitened traits.”*

(3) In the simulation studies, I want to see how the extent of LD affects our assessment precision on the pervasion of pleiotropy. You may need to simulate more scenarios by allowing LD to vary in a wide range.

*Authors reply: Our simulations fixed the LD architecture to the architecture observed in the 1,000 Genomes European population, so our simulations do not include a parameter controlling the extent of LD. However, as the Reviewer noted, there are of course regions within the genome that have higher or lower levels of observed LD. We have now examined the difference between these regions in our simulations. Consistent with our expectation, we find that regions of higher LD substantially inflate our estimates of pleiotropy, and that our correction for LD restores these estimates to a level similar to simulations where no LD is present.*

*Manuscript changes:*

*We have added a figure showing this analysis as Supplementary Figure 1.*

*We have added the following text to the Results section, under “Power to detect pleiotropy in simulations:”*

*To introduce LD in the simulations, we used real LD architecture from 800000 SNPs from 1000 Genomes European population. We simulated Z-scores independently for each SNP and then propagate LD for a given SNP by “contaminating” its Z-score according to the Z-scores of the SNPs in LD with it.*

*,,, and that the LD correction dramatically reduces the dependence of the pleiotropy score on LD (Supplementary Figure 1).*

(4) In addition to LD, the linkage between different loci may also contribute to pleiotropy. How can you study the contribution of the linkage?

*Authors reply: The correction we have applied for LD appears to correct adequately for the effect of linkage between loci (see Response to Comment 3 above). Additionally, we see minimal differences between using all loci to compute our scores and using LD-pruned loci to compute our scores. We believe that we have accounted for the effect of linkage on the pleiotropy scores.*

(5) The authors studied 372 phenotypes to test their framework. I am interested to see what these phenotypes are? Are they correlated? It makes more sense to give the correlation table of these phenotypes.

*Authors reply: We have now made publicly available the set of 372 phenotypes and their correlations on our GitHub page at https://github.com/rondolab/PleiotropyScore. This is listed in the manuscript under “Availability of data and material.” We have also added a link to the GitHub page to download the correlation matrix directly:*

*“The Zscore correlation matrix for the 372 UK Biobank phenotypes is directly downloadable here: https://github.com/rondolab/PleiotropyScore/blob/master/inst/shiny-examples/PleiotropyScoreApp/www/UkBiobank_Zscore_CorrelationMat.xlsx”*

(6) According to the definition of horizontal pleiotropy, BMI and body height should not be explained to be under the control of horizontal pleiotropy. BMI is a composite trait of height and body mass. The relationship between BMI and height should be explained as something else. Among these 372 traits, how many have relationships like BMI and height?

*Authors reply: As the Reviewer noted, these are the traits that are most influenced by highly pleiotropic variants. We would like to clarify that we do not necessarily expect traits strongly associated with horizontally pleiotropic variants to have no vertical pleiotropic relationships among themselves. Indeed, we should expect highly pleiotropy-associated traits to be highly correlated to each other, since they are all under the control of the same highly pleiotropic variants. We have added text to clarify this important point.*

*Manuscript changes: We have added text to the Results section, under the subheading “A quantitative score for pleiotropy.” It now reads in total as follows:*

*To characterize the phenotypic associations of these loci, we used our replication dataset of published GWAS summary statistics for 73 human quantitative traits and diseases, plus nine additional traits we excluded from our replication dataset for a total of 82 (Methods). We are not able to compute directly the degree of pleiotropy exhibited by these traits, since our definition of horizontal pleiotropy applies only to individual variants and does not apply to traits. However, we can identify traits whose GWAS variant associations are correlated to our pleiotropy score, which in some sense represents the traits that contribute most to our signal of pervasive horizontal pleiotropy. Figure 6c shows the correlations between our LD-corrected pleiotropy score (P_m^LD and P_n^LD) and the association statistics for these 82 traits and diseases. The most strongly correlated traits were anthropometric traits like body mass index, waist and hip circumference, and height; certain blood lipid levels, including total cholesterol and triglycerides; and schizophrenia. These are all known to be highly polygenic and heterogeneous traits. The least correlated traits include several measurements of insulin sensitivity and glucose response, such as the insulin sensitivity index (ISI), certain features of brain morphology, and the inflammatory biomarker lipoprotein(a). This may be partly due to low sample size of the corresponding GWASs. However, these correlations do not appear to be driven exclusively by sample size: in cases where multiple GWASs for the same trait have been performed on subsamples of the population (for example, males only, female only, and combined), the sample size only marginally affects the correlation (Supplementary Table 4). Another contributing factor may be heritability: height, in particular, is among the most heritable traits we examined, while ISI and the brain morphology features are among the least.*

*We have edited the legend for Figure 6c (formerly Figure 4c) to read as follows:*

*Panel c shows the contribution of pleiotropic variants to 82 complex traits and diseases. Contribution of pleiotropic variants is calculated as the correlation coefficient between the absolute value of Z-scores and the pleiotropy score among variants that are genome-wide significant for the pleiotropy score (P < 5 × 10-8 for P_m^LD and P_n^LD respectively).*

In summary, this is a nice work and should be published after addressing the above comments.

*Authors reply: We thank the Reviewer for their helpful and positive comments.*

*Other Minor Changes*

*We reran the polygenicity-corrected analysis, and have modified the text in several places to reflect this, as well as including updated versions of Supplementary Figures S7-S8. The most significant difference is that there are now loci with genome-wide significant P_n^LD scores.*

*Manuscript changes: We have edited the paragraph in the Results section under the subheading “Genome-wide pleiotropy study identifies novel biological loci.” to include both components of the pleiotropy score instead of just P_n. It now reads, in full:*

*By analogy to standard GWAS, our GWPS methodology can identify individual variants that have a genome-wide significant level of horizontal pleiotropy. Using the LD-corrected magnitude score P_m^LD, we identified 74,335 variants in 8,093 independent loci with a genome-wide significant level of horizontal pleiotropy, while using the LD-corrected number of traits score P_n^LD identified 18,393 variants in 2,859 independent loci with a genome-wide significant level of horizontal pleiotropy, all of which are also identified by the LD-corrected magnitude score P_m^LD (Methods, Supplementary Table 2). Applying the same analysis to the Polygenicity/LD-corrected pleiotropy score, using the Polygenicity/LD-corrected magnitude score P_m^P identified no genome-wide significant loci, but using the Polygenicity/LD-corected number of traits score P_n^P identified 2,674 variants in 432 loci. Strikingly, a majority of loci significant in P_n^LD (1,519 of 2,859) or P_n^P (294 of 432), along with a sizeable minority of loci significant in P_m^LD (2,934 of 8,093), have no entry in the NHGRI-EBI GWAS catalog, meaning that they have never been reported as an associated locus in any published GWAS. These loci represent an under-recognized class of genetic variation that has multiple weak to intermediate effects that are collectively significant, but no specific strong effect on any one particular trait. Functional enrichment analysis on genes near these genome-wide significant loci implicates a wide range of biological functions, including cell adhesion, post-translational modification of proteins, cytoskeleton, transcription factors, and intracellular signaling cascades (Additional File 2). Loci significant in P_n^P show a more focused subset of functions, with a greater role for nuclear proteins regulating transcription and chromatin state, suggesting that these are the functions that exhibit pleiotropy beyond the baseline level induced by polygenicity. The role of these novel loci and these biological processes in human genetics and biology may be a fruitful area for future study, with the potential for biological discovery.*

*We have made a minor change to the title. It now reads:*

*Pervasive horizontal pleiotropy in human genetic variation is driven by extreme polygenicity of human traits and diseases*

**Second round of review**

**Reviewer 1**

My concerns have now been adequately addressed.
